# Stroke Genomics: Current Knowledge, Clinical Applications and Future Possibilities

**DOI:** 10.3390/brainsci12030302

**Published:** 2022-02-23

**Authors:** Sandeep Appunni, Muni Rubens, Venkataraghavan Ramamoorthy, Anshul Saxena, Peter McGranaghan, Emir Veledar

**Affiliations:** 1 Government Medical College, Kozhikode 673008, India; sandeepappunni@gmail.com; 2 Baptist Health South Florida, Miami, FL 33176, USA; drvenky37@gmail.com (V.R.); anshuls@baptisthealth.net (A.S.); 3 Department of Internal Medicine and Cardiology, Charité—Universitätsmedizin Berlin, Corporate Member of Freie Universität Berlin and Humboldt Universität zu Berlin, 10117 Berlin, Germany; emirv@baptisthealth.net

**Keywords:** stroke, genomics, Mendelian inheritance, GWAS, PheWAS

## Abstract

The pathophysiology of stoke involves many complex pathways and risk factors. Though there are several ongoing studies on stroke, treatment options are limited, and the prevalence of stroke is continuing to increase. Understanding the genomic variants and biological pathways associated with stroke could offer novel therapeutic alternatives in terms of drug targets and receptor modulations for newer treatment methods. It is challenging to identify individual causative mutations in a single gene because many alleles are responsible for minor effects. Therefore, multiple factorial analyses using single nucleotide polymorphisms (SNPs) could be used to gain new insight by identifying potential genetic risk factors. There are many studies, such as Genome-Wide Association Studies (GWAS) and Phenome-Wide Association Studies (PheWAS) which have identified numerous independent loci associated with stroke, which could be instrumental in developing newer drug targets and novel therapies. Additionally, using analytical techniques, such as meta-analysis and Mendelian randomization could help in evaluating stroke risk factors and determining treatment priorities. Combining SNPs into polygenic risk scores and lifestyle risk factors could detect stroke risk at a very young age and help in administering preventive interventions.

## 1. Introduction

Several risk factors and complex pathways are involved in the pathophysiology of stroke. Stroke is the second leading cause of death worldwide after heart attack [1]. The human genome project has helped in understanding many genetic factors that are associated with stroke [2,3,4]. Several studies have reported genetic predisposition to stroke in both human beings and animal models. However, the definition of genetic risk factors for stroke is not well established. Since no single specific gene has been responsible for stroke, it has been hypothesized to be a multifactorial polygenic disorder [5]. In this study, we used a narrative review method to understand the current advances, clinical applications, and future possibilities of the associations between genetic factors and stroke.

## 2. Genetic Factor Associated with Stroke (Non-Modifiable Factors in Stroke)

Several studies, such as the classical twin study which consisted of 15,924 twin pairs have been designed to assess the genetic factors associated with stroke [6]. Likewise, another twin study provided evidence for genetic factors that may increase the risk of stroke related events, such as death and hospitalization [7]. This study found greater concordance rates for these associations among monozygotic twins, compared to dizygotic twins [7]. These two studies were designed long before the human genome project. There could be different environmental effects affecting the results of these studies, which was a major limitation [8]. Previous studies have reported that first degree relatives are at an increased risk for stroke [9]. The preponderance of large and small vessel strokes, compared to cardioembolic strokes, is higher among subjects with a family history of stroke [9]. Sex is an important factor to influence stroke outcome indicating the possible role of the sex chromosome and associated genes; however, recently a review reported no association between sex and stroke [10]. Similarly, though ethnicity is not widely considered as an important factor affecting acute stroke outcome; it may influence the long-term outcome [10,11]. A recent study identified that levels of lipoprotein-A were significantly associated with adverse stroke outcomes, and were substantially higher in the Black, compared to the White population [12]. In addition, hematological disorders are responsible for nearly 1.3% of acute stroke. Some of the common hematological disorders associated with stroke include polycythemia vera, sickle-cell disease, Waldenström macroglobulinemia, multiple myeloma, essential thrombocythemia, thrombotic thrombocytopenic purpura, protein C deficiency, Protein S deficiency, antithrombin deficiency, and Factor V Leiden. A substantial number of these disorders have a genetic predisposition. For example, a large proportion of polycythemia vera patients have a mutation in the exon 14 of the *JAK2* gene (*JAK2V617F*), whereas a smaller proportion has mutations in the *JAK2* exon 12 [4].

### 2.1. Heritability Genes in Stroke (Monogenic and Polygenic Inheritance in Stroke Etiology)

Several animal model studies were conducted to identify potential candidate genes associated with stroke outcome. These studies analyzed the association of single nucleotide polymorphisms (SNPs) in targeted genes. The SNP of COX-2 and rs20417 genes were associated with early neurological deterioration [13,14]. However, these studies are not supported with further replicational studies and hence warrant further in-depth research. A study reported that several single-gene disorders might influence stroke, such as sickle cell disease, Fabry’s disease, homocystinuria, mitochondrial myopathy, and encephalopathy [15]. A rare stroke case caused by mutations in the Notch 3 gene (OMIM*600276) showed heritable patterns [16], which was also reported as a single-gene disorder. Cerebral Autosomal Dominant Arteriopathy with Subcortical Infarcts and Leukoencephalopathy (CADASIL) caused by different types of mutations of Notch 3 gene are associated with extensive cerebral small vessel damage, marked by the accumulation of granular osmiophilic material (GOM) [17]. Molecular evaluation of the vascular smooth muscles in CADASIL patients showed increased oxidation of soluble guanyl cyclase associated with decreased cyclic GMP levels, which impaired vasorelaxation of the cerebral vasculature [17]. A number of molecular pathways associated with cell adhesion, extracellular matrix components, misfolding control, autophagy, angiogenesis, and transforming growth factor β (TGFβ) signaling pathway are altered in CADASIL. Metabolic impairment, such as diabetes mellitus further expedites the pathological damage to the cerebral small blood vessels in Notch 3 mutation, resulting in endothelium mitochondrial dysfunction and vascular basement membrane injuries [18]. This suggests that the heritability of Notch 3 mutation increases the risk for ischemic stroke from small vessel diseases, such as CADASIL (Table 1).

Heterozygous mutations in the 3ʹuntranslated region (UTR) of the collagen 4A1 encoding gene may also influence ischemic stroke [19]. A glycine substitution mutation in the triple-helical domains of COL4A1 and COL4A2 may develop neurological and non-neurological manifestations, including hemorrhagic stroke [20]. The genomic data enables accurate analysis of heterozygous mutations. Another study identified heterozygous mutations in High-Temperature Requirement Serine protease A1 (HTRA1) encoding gene that manifest as stroke and cognitive decline in people aged more than 45 years [21]. Other mutations were also identified in the HTRA1 gene that may cause cerebral autosomal recessive arteriopathy in younger people who are between 10 to 30 years of age [22]. Similarly, mutations in adenosine deaminase 2 (ADA2), cathepsin A (CTSA) and forkhead-box C1 (FOXC1) genes were also found to be associated with autosomal dominant small vessel disease [23,24,25]. In addition, there are several other candidate genes under investigation for a possible association with stroke.

### 2.2. Multifactorial Stroke and SNPs

It is challenging to identify individual causative mutations in a single gene because many alleles are responsible for minor effects. Therefore, multiple factorial analyses using SNPs were used to gain newer insight by identifying potential genetic risk factors. For example, a study by Mola-Caminal et al. identified a locus located within a candidate gene [26], which can help in understanding the genetic mechanisms involved in stroke. Newer variants in the gene pals1-associated tight junction (PATJ) were linked to poor functional outcomes at 3-month post-stroke [26]. rs76221407 was the major SNP variant of the PATJ gene, which was associated with poor outcomes in stroke subjects after 3 months. The locus STRK1 was mapped to identify a susceptible gene for stroke for the first time [27]. Another study identified a strong association between the phosphodiesterase 4D gene (PDE4D; OMIM 600129*) and two major subtypes of stroke, cardiogenic and carotid stroke. Among 260 PDE4D gene SNPs, six were found to be significantly associated with stroke. Some of the SNPs were from UTR; therefore, these SNPs may affect the transcription of PDE4D [28]. The 5-lipoxygenase activating protein gene (ALOX5AP; OMIM 603700*) was also associated with an increased risk of stroke [29]. ALOX5AP SNP haplotypes increase the production of leukotriene B4 in stimulated neutrophils, thereby contributing to vascular inflammation in myocardial infarction and stroke [29]. The main limitation of studying candidate genes for SNPs and their association with stroke is that they are time consuming and require significant resources [30,31], and could be associated with false positive results.

## 3. Genomic Evaluation in Stroke

Several studies were designed during the 1990s to observe the effect of Mendelian genetics and candidate genes on stroke [32]. Subsequently, the human genome project enabled accurate SNP analysis by using the Genome-Wide Association Study (GWAS) [33].

### 3.1. Genome-Wide Association Study (GWAS) in Stroke

The first GWAS in stroke, Ischemic Stroke Genetics Study (ISGS) which included 250 patients and controls, was published in 2007 [34]. This study failed to identify any genetic locus, which was explicitly associated with stroke. Subsequently, studies focused on a specific region of chromosome 9 (9p21.3) and found an association with stroke [35]. This region was associated with coronary heart disease [36], and hence it was suggested that heart disease and ischemic stroke share similar polymorphisms. Another research group also studied chromosome 9 and found modest associations between ischemic stroke and variants (rs2383207 and rs10757274) of the 9p21 region [37]. Finally, six SNPs were identified, including rs2383207 in the 9p21 region, which were independently associated with the ischemic stroke (large artery atherosclerotic subtype) [38]. This suggests that chromosome 9p21 is an important risk locus that shares SNP variants that are common for both ischemic stroke and coronary artery disease. 

A case-control study found a significant association between the 4q25 region and the cardioembolic subtype of ischemic stroke [39]. This region was also associated with all types of ischemic stroke, though to a lesser degree [39]. This study found that markers of atrial fibrillation, such as rs2200733 and rs10033464, have a strong association with ischemic stroke by increasing the risk for cardioembolic events. Another locus, the 16q22 was also found to be associated with cardioembolic stroke [40]. GWAS also found robust associations between intracranial aneurysms and loci on 2q, 8q, and 9p21 regions [41,42] The first prospective GWAS on stroke was the Heart and Aging Research in Genomic Epidemiology (CHARGE) study, which included 19,600 participants with 1544 strokes incidence [43]. This study identified two SNPs (rs11833579 and rs12425791) in the 12p13 region of chromosome 12 and within 11 kb upstream of the gene NINJ2 (Ninjurin 2), all of which were significantly associated with stroke. 

The GWAS projects for ischemic stroke have identified many SNPs that are associated with stroke [44,45,46,47,48,49,50,51,52,53,54]. Among them, one study identified variants associated with different subtypes of stroke. This study showed that variants close to PITX2 (paired like homeodomain 2) and ZFHX3 (zinc finger homeobox 3) were linked to cardioembolic stroke. Variants on chromosome 9p21 locus and a novel variant on chromosome 7p21.1 within the histone deacetylase 9 (HDAC9) gene were associated with large vessel stroke [53]. This study suggested that genetic heterogeneity was associated with different stroke subtypes and would further demand subtype-specific studies for understanding genetic alterations in ischemic stroke.

Several GWAS consortia have been using and analyzing extensive datasets from major national and international projects. For example, SiGN project contains 14,549 cases from 24 genetic research centers located in the United States (*n* = 13) and Europe (*n* = 11) [55]. The MEGASTROKE consortium analyzed multi-ancestry GWAS data from more than 67,000 stroke cases and 454,000 controls and identified 32 significant loci to be associated with stroke [56]. Among them, two loci were independently associated with large artery stroke, and one with cardioembolic stroke. However, GWAS data has provided different associations between genes and stroke among different population and ethnic groups. For example, variants of the apelin receptor gene (APLNR, rs9943582) were associated with increased risk of ischemic stroke among the Japanese population, while these variants had no association with stroke among the Chinese Han Population [57]. Similarly, GWAS identified that rs2107595 SNP in the HDAC9 gene was associated with large-vessel ischemic stroke among the European population, while not among the Chinese Han population [58]. Another GWAS identified an SNP locus on region 10q25.3 of chromosome 10 (rs11196288) to be associated with the risk of early-onset ischemic stroke among the European population [59]. However, this SNP locus showed different susceptibility levels among the Chinese Han population. Similarly, in another study, there were differing associations between Caucasians and Chinese Han populations with respect to the relationship between SNPs of rs2200733 and rs6843082 on chromosome 4q25 and stroke [60]. These SNPs were associated with ischemic stroke among Caucasians, but not among the Chinese Han population. These varying results suggested that genetic factors are also modulated by other racial and ethnic factors and could provide unclear results. Therefore, it is recommended that GWAS data should be analyzed after population based sub-grouping.

### 3.2. GWAS and Comorbidities of Stroke

GWAS not only identified the genetic basis for stroke but also associations between genetic factors and comorbidities for stroke. The most important comorbidity associated with stroke is hypertension and is responsible for 30–40% of population-attributable risk for stroke [61]. Other comorbidities associated with stroke are smoking, diabetes, atrial fibrillation, and coronary heart disease. Several loci have been identified which have an association with stroke and its comorbidities. For example, 4q25 region of chromosome 4 [39] and a variant of the ZFHX3 gene in the 16q22 region of chromosome 16 [40] were associated with ischemic stroke and atrial fibrillation, 9p21 region was associated with stroke and diabetes [62], and serine/threonine kinase gene (STK39) variants were associated with stroke and hypertension [63]. The MEGASTROKE consortium identified a total of 32 loci that were significantly associated with stroke. Among them, five were associated with blood pressure, five with coronary heart disease, two with low-density lipoprotein (LDL) cholesterol, two with atrial fibrillation, two with venous thromboembolism, one with white matter hyperintensities, and one with carotid plaque [56]. This study identified a strong association between coronary heart disease and large artery stroke as well as blood pressure and all stroke subtypes. This study also found that cardioembolic stroke and large artery stroke, though not small vessel stroke, were associated with venous thromboembolism. However, an interesting finding from this study was that high-density lipoprotein (HDL) cholesterol was inversely associated with small vessel stroke [56]. Another study reported that the rs4376531 variant among diabetes predicted the risk for atherothrombotic stroke [64]. Another GWAS study found that C-reactive protein gene polymorphisms increased its synthesis level, which in turn increased the risk for stroke [65]. 

### 3.3. Genomic Determinants of Stroke Outcomes

Researchers started using data from GWAS for identifying genetic determinants of stroke outcomes only recently [2]. A genome wide meta-analysis (GWMA) of 12 stroke cohorts identified that the Pals1-associated tight junction (PATJ) variant was significantly associated with adverse functional outcomes after three months of stroke [26]. However, the molecular mechanism of how the variants of the PATJ gene led to these outcomes is still unclear. Mola-Caminal et al. reported that the major variant rs76221407 in the PATJ gene was a key genotypic trait associated with poor functional outcomes after three months of stroke onset [26]. Another GWMA study identified the SNP rs184681 to be significantly associated with functional outcomes of neural plasticity between 60 and 190 days after stroke onset [66]. In another study, the genetic imbalance was associated with unfavorable outcomes after 2–6 months of stroke, after adjusting for age, sex, race, and stroke subtypes [67]. 

## 4. Preclinical Studies Supporting Genomic Analysis in Stroke

It is challenging to study preclinical stroke models, although several animal studies have been designed to overcome the effects of host genetic variations. However, even after genetic background restriction, studies using animal models have been questioned for assessing complex polygenetic disorders, such as stroke [68]. Nevertheless, animal models are still valuable to study basic mechanisms and factors, such as environmental and dietary factors related to stroke. In addition, animal studies have been used for identifying potential targets involved in inflammatory signaling of stroke outcomes among humans [69].

Studies have shown that Metastasis-Associated Lung Adenocarcinoma Transcript 1 (MALAT1) expression could be induced in vitro in endothelial cells undergoing oxygen-glucose deprivation (OGD) [70,71]. Transcriptional downregulation of MALAT1 in OGD induced primary mouse brain microvascular endothelial cells led to overexpression of pro-apoptotic factor bim and increased pro-inflammatory cytokines, such as MCP-1, IL-6, and E-selectin [72]. Moreover, in vivo MALAT1 knockout mice showed severe neurological deficits, compared to wild-type controls in response to transient focal ischemia [72]. Additionally, other studies have demonstrated that MALAT1 promotes endothelial cell survival, angiogenesis, and vascular integrity in stroke [73,74,75,76,77]. MALAT1 plays a crucial role in regulating post-stroke pathophysiology; however, further studies are required to understand the contexts and conditions under which MALAT1 mediates beneficial versus deleterious outcomes.

Upregulated maternally expressed gene (MEG3) in the mouse brain and primary neurons were linked to increased cell death in cerebral ischemia [78,79,80]. Long non-coding RNA (lncRNA) MEG3 functions as a competing endogenous RNA (ceRNA) and binds to miR-21 and downregulates the miR-21/PDAC pathway, leading to neuronal death in ischemic neurons [80]. miR-21 overexpression reverses the effect of OGD reperfusion induced neuronal apoptosis in vitro. Another investigation showed that downregulation of MEG3 was associated with increased micro-vessel density in rat neurons [81]. Therefore, MEG3 exhibits differential expression after stroke among different species and cell types while downregulation of MEG3 is strongly associated with post-stroke neuroprotection.

The Small Nucleolar RNA Host Gene 12 (SNHG12) expression after ischemic injury was increased both in vitro and in vivo [82,83,84]. The N2a cell line and mouse primary hippocampal neuronal study has shown higher expressions of lncRNA SNHG12 among neuronal cells undergoing ischemia [83], while downregulation of miR-199a by SNHG12 decreases cell death and inflammation [82]. lncRNA SNHG12 improves neuronal survival following OGD reperfusion induced ischemia through miR-199a downregulation by sirtuin-1 upregulation and activation of adenosine 5′ monophosphate-activated protein kinase (AMPK) pathway [83]. This suggests that increased expression of lncRNA SNHG12 salvages injured ischemic neurons. During transient ischemia, circulating H19 levels become higher in blood and brain among stroke patients. Experimental mouse model studies have shown that knockdown of H19 could decrease edema, infarct volume, and neurological deficits after stroke [85,86]. Though several studies have looked for the role of lncRNA in modulating the post-stroke pathophysiology, only a few have explored the genetic variations of lncRNA and altered expression among stroke patients [85,87,88,89,90,91,92]. Overall, these studies introduce the possibility that the evaluation of lncRNA expression or lncRNA gene loci could be a useful clinical tool for assessing the risk for developing stroke.

## 5. Extending Genome-Based Evaluation into the Clinical Scenario

The global prevalence of stroke is consistently increasing, and there are limited therapeutic interventions. Therefore, developing advanced treatment strategies to manage stroke and post-stroke brain damage is important. Several studies have already completed preliminary research to integrate genetic data into routine clinical practice and precision medicine. Extending genome-based studies could help in developing therapeutic and predictability capabilities in managing the early stages of stroke.

### 5.1. Stroke Risk Prediction in Childhood

Several studies were designed for the identification of stroke by gene expression profiling. Studies have predicted ischemic stroke with as high as 80% accuracy through analysis of a panel of 22 genes from peripheral blood mononuclear cells (PBMC) [93,94]. Using the latest technology and developments from genetic data, high-risk individuals could be identified by applying polygenic risk scores for common genetic variants, even during childhood [95,96]. These methods can enable the opportunities for early prevention of stroke. Recently, a study developed a polygenic risk score derived from a panel of 90 SNPs to identify individuals with 35% increased risk for stroke [97]. Risk scores for stroke based on lifestyle factors, such as smoking, diet, body mass index (BMI), and physical activity have shown that lifestyle risks were similar across all polygenic risk score strata.

Recently, studies have applied Mendelian randomization to identify risk for stroke [98]. For example, a mendelian randomization study identified factors, such as BMI and waist-to-hip ratio, in order to identify individuals with greater risk for ischemic stroke [99]. The differential effects of LDL and HDL on cardioembolic stroke, small vessel stroke, and large artery stroke observed in the MEGASTROKE consortium study were also confirmed by a Mendelian randomization study [100]. Similarly, differential effects of type 2 diabetes were observed for different etiological stroke subtypes by the Mendelian randomization studies [101,102]. Mendelian randomization studies could be further applied for identifying novel risk factors for stroke. With the increasing availability of genomic data, Mendelian randomization studies will become more relevant and applicable in clinical practice. Although additional research is required to evaluate and improve genetic risk prediction of stroke, these studies highlight the potential for early risk stratification and prevention of stroke via genetic evaluation.

### 5.2. Exploration of Potential Therapeutics

Currently, the pharmacological treatment of stroke is mainly based on recombinant tissue plasminogen activator (rtPA). rtPA was developed based on genetic data. Nevertheless, pharmacological treatment strategies for stroke have not significantly progressed over the years. The FDA first approved RNA-targeting antisense oligos therapy for spinal muscular atrophy in 2017. After that, in 2018, FDA approved the first RNAi therapy as a treatment option for peripheral nerve disease. Increasing transthyretin in tissues is the main reason for this disease and is caused by hereditary transthyretin-mediated amyloidosis [103]. The exploration of this genetic target could significantly expand the pharmacological applications of stroke treatment in the future.

### 5.3. Exploiting Genetics for Potential Drug Discovery

Genomic data offers a great potential for drug development of stroke by identifying causal pathways and drug targets and could determine the safety and efficacy of pharmacological interventions [104,105,106]. These approaches were developed to personalize the dose and minimize the side effects. Mendelian randomization studies [107] and other studies have shown the use of protective variants [108,109,110] and have demonstrated naturally occurring human knockouts for phenotypic effects on stroke outcomes [111]. Currently, phenome-wide association studies (PheWASs) show promise and have analyzed large datasets with detailed genotyping and phenotyping data with multiple traits [112,113]. Therefore, using genetic and phenetic data for potential drug discovery and precision medicine is now an advancing and emerging major research focus.

## 6. Future Directives

Several genetic and genomic factors have already been identified for stroke and some overlap with comorbidities as previously described (Table 1). Many of these studies, such as those associated with vascular risk, monogenic vasculopathies, the leukotriene pathway, and other GWAS require further detailed investigation. Although vasculopathy of CADASIL was not associated with heart diseases, higher rates of myocardial infarction [114] and unexplained sudden deaths [115] among these patients require additional investigations. 

Currently, there are several ongoing GWAS and PheWAS with large sample sizes for identifying newer and undiscovered loci associated with stroke [56]. Biobanks and databases will enormously expand the opportunity for gene discovery [116], and thereby, accelerate the progress in this field. However, data from non-European ancestry is inadequate. Therefore, for studies to be effective across all populations, ancestry-specific genetic data should be developed. The development of prospective drugs has now become much easier after GWAS and PheWAS for several common diseases [104]. However, further, improvement is required to develop novel cell and tissue models to study functional genomics and multilevel omics of stroke. Nevertheless, genetic studies focusing on treatment and recovery after stroke are in their infancy and require much more details for clinical applications [26,117]. Strokes could lead to significant cognitive decline and vascular dementia because of cerebral small vessel diseases. However, there is very little data from studies estimating the heritability of cerebral small vessel diseases. A growing body of evidence from epidemiological and genetic studies suggests that early cerebral small vessel diseases are heritable. It is, therefore, imperative that future studies should address the genetic factors associated with cerebral small vessel disease, as well as the potential clinical outcomes, to assess the genomics of vascular cognitive decline.

## 7. Limitations

Though we have reviewed several genetic factors associated with stroke, several others that have not been covered in this review require additional exploration. We have primarily focused on genetic factors that are adversely associated with stroke. There are some protective genetic factors as well which need further exploration. Though we have explored genetic factors associated with stroke, there are epigenetic factors that need additional evaluation. In addition, we could not explore in detail how genetic factors could be incorporated in precision medicine and how genetic data could be integrated with other omics data, such as proteomic, metabolomic, and transcriptomic data since they are beyond the scope of this review. 

## 8. Conclusions

The prevalence of stroke and the global burden remains high. Therefore, discovering genetic variants and biological pathways offer has revived hopes for novel therapeutics, drug targets, and effective interventions. Genetic information can be used to improve stroke diagnosis and prognosis. Several GWAS and PheWAS have identified many independent loci associated with stroke which could be instrumental in developing newer drug targets and novel therapies. The application of analytical techniques, such as meta-analysis and Mendelian randomization could also facilitate evaluating risk factors and stroke outcomes and prioritizing potential therapeutic targets. Accumulating SNPs into polygenic risk scores and combining them with lifestyle risk factor scores could enable the possibility of identifying individuals who are at a greater risk for stroke even at a younger age.

## Figures and Tables

**Table 1:** Studies showing stroke related events and clinical or pathological outcomes.

Author, Year	Stroke Related or Associated Events	Outcome (Clinical or Pathological)
Bak et al., 2002	Higher stroke death and hospitalization in MZ compared to DZ twins	Potential role of genetic factors in stroke etiology
Flossmann et al., 2004	Large and small vessel stroke in comparison to cardioembolic stroke	Positive family history enhances the risk of large and small vessel stroke
Neves et al., 2021	CADASIL with gain of function mutation of notch 3	Enhanced cerebral small vessel disease marked by GOM
Neves et al., 2021	CADASIL and impaired cerebral vasorelaxation	Augmented soluble guanyl cyclase oxidation and reduced cGMP
Felczak et al., 2021	Diabetes mellitus and cerebral small blood vessel injury in notch 3 mutation	Associated with mitochondrial dysfunction in endothelial cells and vascular basement membrane injury
Mola-Caminal et al., 2019	PATJ variants	Poor functional outcome post-stroke
Helgadottir et al., 2004	Vascular inflammation triggered by 5-lipoxygenase activating protein gene variants	Increase the risk for myocardial infarction and stroke
Smith et al., 2009	Genetic determinants for ischemic stroke on chromosome 9p21 shared with coronary artery disease	2 common variants, rs2383207 and rs10757274 associated with modest increase in ischemic stroke risk
Ikram et al., 2009	SNPs (rs11833579 and rs12425791) in chromosome 12p13	Increased risk for ischemic stroke
Gretarsdottir et al., 2008	Atrial fibrillation associated cardioembolic events	Increased risk for ischemic stroke associated with markers rs2200733 and rs10033464 located on chromosome 4q25
Malik et al., 2018	32 loci associated with ischemic stroke and its subtypes	Shared traits with blood pressure, cardiac abnormalities, LDL cholesterol, atrial fibrillation and venous thromboembolism
Mola-Caminal et al., 2018	Top variant rs76221407 in PATJ gene	Associated with poor functional outcome in ischemic stroke
Zhang et al., 2017	Downregulation of MALAT1 expression in in vitro and in vivo stroke model	Enhanced pro-apoptotic bim and pro-inflammatory cytokines (MCP-1, IL-6, and E-selectin) in vitro. Post-stroke functional deterioration in vivo.
Yan et al., 2017	OGD-reperfusion induced neuronal injury in vitro	LncRNA MEG3 induced neuronal death by downregulating miR-21/PDAC pathway
Long et al., 2018	LncRNA SNHG12 overexpression following neuronal ischemia	Suppress neuronal death by downregulating miR-199a
Yin et al., 2019	LncRNA SNHG12 salvages ischemia injured neurons	Upregulated Sirtuin-1 and activated AMPK pathway in vitro

Abbreviations: MZ, monozygotic; DZ, dizygotic; CADASIL, cerebral autosomal dominant arteriopathy with subcortical infarcts and leukoencephalopathy; GOM, granular osmiophilic material; cGMP, cyclic guanosine monophosphate; PATJ, pals1-associated tight junction; SNP, single nucleotide polymorphism; LDL, low density lipoprotein; MALAT1, metastasis-associated lung adenocarcinoma transcript 1; MCP-1, monocyte chemoattractant protein-1; IL-6, interleukin-6; OGD, oxygen-glucose deprivation; LncRNA, long non-coding RNA; MEG3, maternally expressed gene 3; miR-21/PDAC, micro ribonucleic acid-21/pancreatic ductal adenocarcinoma; SNHG12, small nucleolar RNA host gene 12; miR-199a, micro ribonucleic acid-199a; AMPK, adenosine monophosphate-activated protein kinase.

## Data Availability

Not applicable.
